# Allogenic MSC infusion in kidney transplantation recipients promotes within 4 hours distinct B cell and T cell phenotypes

**DOI:** 10.3389/fimmu.2024.1455300

**Published:** 2024-10-09

**Authors:** Sanne H. Hendriks, Sebastiaan Heidt, Marlies E.J. Reinders, Frits Koning, Cees van Kooten

**Affiliations:** ^1^ Department of Immunology, Leiden University Medical Center, Leiden University, Leiden, Netherlands; ^2^ Department of Internal Medicine, Nephrology and Transplantation, Erasmus MC Transplant Institute, Erasmus University Medical Center, Rotterdam, Netherlands; ^3^ Department of Internal Medicine (Nephrology) and Transplant Center, Leiden University Medical Center, Leiden University, Leiden, Netherlands

**Keywords:** kidney transplantation, immunosuppression, mesenchymal stromal cells, immune regulation, mass cytometry, allogenic

## Abstract

**Background:**

Infusion of mesenchymal stromal cells (MSCs) has been proposed as immune-modulatory therapy in solid organ transplantation. The use of allogenic MSCs could improve standardization and allow for direct availability of the product.

**Method:**

The nonrandomized phase Ib Neptune clinical trial provided safety and feasibility data on the use of allogenic bone-marrow-derived MSCs, infused in 10 patients at week 25 and 26 post kidney transplantation. Here, we performed detailed analysis on the peripheral blood immune cell composition of these patients up to 52 weeks post transplantation. We used a 40 marker antibody panel with mass cytometry to assess potential effects of MSC therapy on the immune system.

**Results:**

We showed minor changes in major immune lineages at week 27, 34 and 52 post kidney transplantation after MSC infusion at week 25 and week 26, confirming previous data with regular flow cytometry. However, in a direct comparison between pre- and post MSC infusion, as soon as 4 hours after MSC infusion, we observed a significant increase in cell numbers of B cell and T cell subsets that shared a unique expression of CD11b, CD11c, CD38, CD39, and Ki-67.

**Conclusion:**

Exploring these CD11b^+^CD11c^+^CD38^+^CD39^+^Ki-67^+^ B cells and T cells in the context of MSC infusion after kidney transplantation may be a promising avenue to better understand the immunological effects of MSC therapy.

## Introduction

Kidney transplantation continues to be the treatment of choice for patients with end-stage kidney disease (1). Short-term kidney graft survival has improved due to, amongst others, the use of potent immunosuppressive agents. However, long-term graft survival has not followed this trend, partially due to long-term toxicity of these immunosuppressive drugs (2–4). For example, calcineurin inhibitors, the backbone of current immunosuppressive regimens, are nephrotoxic and may cause tubulointerstitial damage (5). Therefore, new strategies are being explored to reduce the use of immunosuppressive drugs without increasing the risk of allograft rejection.

The use of mesenchymal stromal cell (MSC) therapy could be such an approach. MSCs have been shown to exert anti-inflammatory, immune-regulatory and tissue repair properties (6, 7). Autologous MSCs have shown beneficial effects in clinical trials in the setting of solid organ transplantation (8–10). However, since *in vitro* expansion to obtain sufficient numbers of MSCs can take several weeks, autologous MSC are not readily available, which is often impractical in the clinical setting. Additionally, the per-patient nature of this process incurs considerable expenses. Alternatively, allogenic MSCs may be used for acute treatments and be beneficial due to standardized quality control and direct availability of the product. Yet, allogenic MSCs could potentially evoke a donor-specific alloimmune response, potentially harming the kidney allograft (11). In our recently published nonrandomized phase Ib Neptune clinical trial, allogenic bone-marrow-derived-MSCs were infused to assess safety and feasibility of administration of third-party MSCs after kidney transplantation (12). In view of safety, the MSCs were selected based on the absence of repeated Human Leukocyte Antigen (HLA) mismatches with the organ donor. The patients received MSCs at week 25 and 26 after transplantation in combination with alemtuzumab induction therapy at day 0 and day 1 and maintenance triple therapy consisting of prednisone, tacrolimus and everolimus. The study showed that the administration of allogenic MSCs was safe and feasible. Additionally, using a major immune lineage flow cytometry panel on freshly obtained blood samples, it was shown that, while monocytes, B cells, NK cells, and CD8^+^ T cells remained stable after the two infusions, CD4^+^ T cells increased upon the infusions. This could potentially be explained by lymphocyte repopulation after induction therapy (12).

Both direct and indirect interactions of MSCs with various immune cells have been described (6, 7, 13–15). However, indirect effects through the release of extracellular vesicles, membrane particles and by undergoing apoptosis are thought to be most relevant due to the short lifespan of MSCs *in vivo (* 6, 7). Our recent work described cell death of MSC within 4 hours of infusion, as shown by the rapid and short-lived appearance of MSC-specific cell-free DNA in the circulation (16). MSC-derived vesicles, including exosomes, that occur during cell death may trigger monocytes and phagocytes to induce tolerogenic dendritic cells and regulatory T cells (Treg) (17, 18). The effects of the MSCs on the peripheral immune cells shortly after intravenous MSC infusion have not been elucidated yet.

Therefore, in the current study we applied mass cytometry to perform in-depth characterization of the peripheral blood immune composition of patients included in the Neptune trial. We exploited a metal-conjugated mass cytometry antibody panel containing 40 antibodies, previously used for a study with autologus MSC therapy (19), for the staining of bio-banked peripheral blood mononuclear cells (PBMCs). We report the influence of MSC therapy in kidney transplantation patients on major immune cell lineages up to 52 weeks after transplantation. Furthermore, we show the short-term effects 4 hours after each MSC infusion at week 25 and week 26 in an in-depth analysis of the immune cell subsets.

## Materials and methods

### Study design

**Figure 1:**
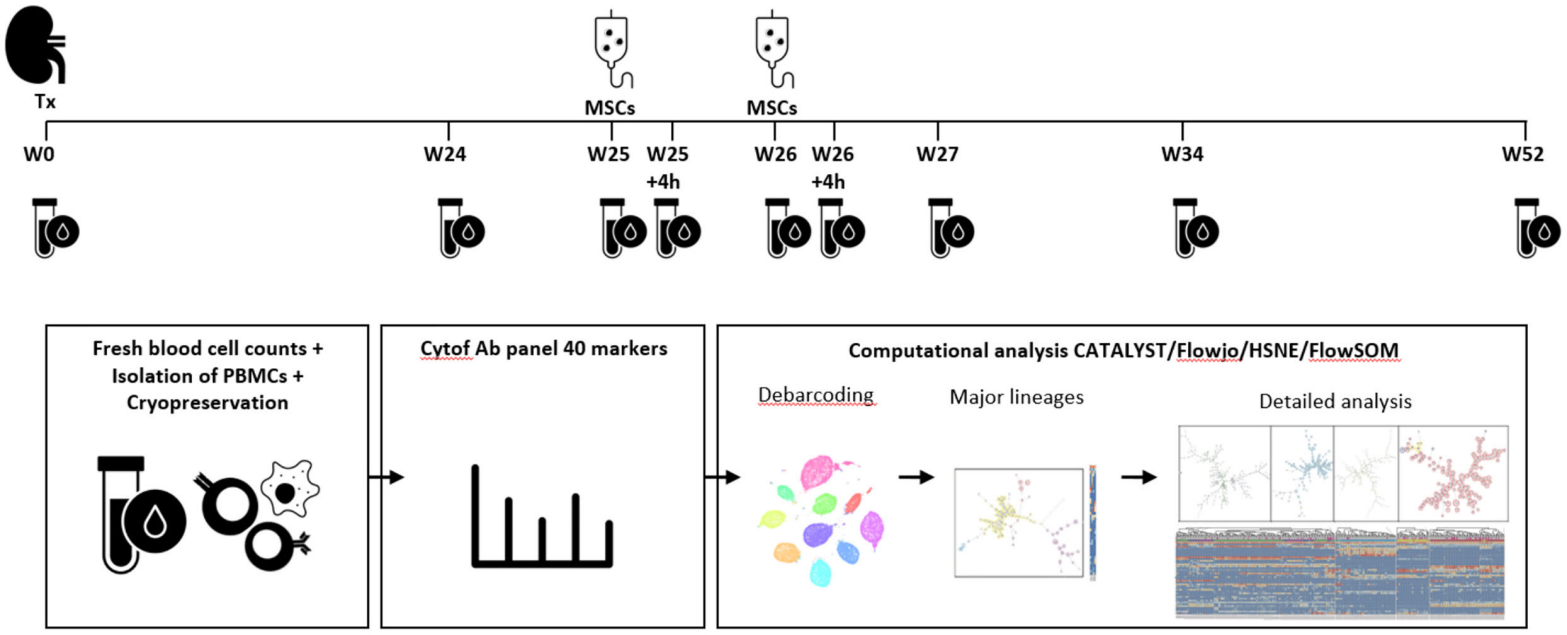
Identification of immune cell lineages in peripheral blood. Experimental setup. MSC infusion took place at week 25 and week 26 after kidney transplantation (Tx). Blood samples were taken at the following time points: week 0, week 24, week 25 before MSC infusion and 4 hours after MSC infusion, at week 26 before and 4 hours after MSC infusion, at week 27, week 34 and week 52. After fresh blood cell counts, isolation of PBMCs and cryopreservation the cells were stained and measured in one batch per patient. After debarcoding, the cells were split based on a first level FlowSOM, already showing a discrepancy between major immune lineages (CD19, CD3, CD16/CD56 and CD11b/CD11c), but a second step provides cleaner data (e.g. removed duplicates) and in-depth analysis which is used for further analysis in this paper.

The Neptune clinical trial was a nonrandomized, prospective, single-center, phase Ib study in living-donor kidney transplant recipients in which allogenic bone marrow derived MSCs were infused 25 weeks and 26 weeks after transplantation (day 0) in 10 patients (**Figure 1**) (12). All patients received alemtuzumab induction therapy at day 0 and day 1 and maintenance triple therapy consisting of prednisone, tacrolimus (Advagraf), and everolimus (Certican). The study was performed at Leiden University Medical Center (LUMC), the Netherlands. The trial design and trial protocol have been previously described and were approved by the local ethics committee at the LUMC, Leiden, and by the Central Committee on Research involving Human Subjects in the Netherlands (12, 20). The trial was performed in accordance with the principles of the Declaration of Helsinki. Inclusion and exclusion criteria were described in the trial protocol (12, 20). Written informed consent was obtained from all participants.

Processing of the MSCs took place at the GMP Facility of the LUMC. The MSC product was infused via peripheral intra venous infusion within a period of 30 min, with a target dose of 1.5 × 10^6^ cells per/kg body weight (range 1– 2 × 10^6^ cells).

During the trial protocol 9 blood samples were obtained of each of the 10 patients; before transplantation (week 0), at week 24, at week 25 and 26 before infusion of MSCs and 4 hours after infusion of MSCs, at week 27, at week 34 and at week 52 (**Figure 1**). All patients received their allocated treatment. One patient had not enough PBMCs stored at 26 weeks.

### Mass cytometry staining and data acquisition

Peripheral blood mononuclear cells (PBMCs) were isolated by Ficoll-Paque density-gradient centrifugation and cryopreserved in liquid nitrogen until time of analysis in 20%FCS, 10%DMSO RPMI. A metal conjugated 40-antibody panel for mass cytometry was developed incorporating all major immune cell lineages. Heavy metal isotope-tagged monoclonal antibodies (mAbs) for mass cytometry are listed in **Supplementary Table S1**. Antibody conjugations and sample staining have been described previously (19). Samples were live-cell barcoded, stained and measured in batches of 9 time point samples and 1 reference sample (samples of each patient were kept within one batch). Barcoding of live cell samples was performed with α-B2M (anti-β-2-microglobulin) and α-CD298 mAbs using a protocol adapted from Mei et al (21). Cells were acquired within 48h of staining on a Helios mass cytometer (Fluidigm) at an event rate of <250 events/sec in Cell Acquisition Solution (Fluidigm) containing 10x diluted EQ Four Element Calibration Beads (Fluidigm). To make a compensation matrix, staining beads (eComp) were individually stained with the conjugated antibodies and incubated for 45 min at a volume of 100µl. After washing, the beads were pooled, washed and acquired in cell staining buffer. Experiments and acquisition were performed in a period of 65 days.

### Mass cytometry data analysis

Data were normalized with EQ-normalization passport for each experiment. Followed by gating to remove debris, dead cells, and doublets with channels 89Y_CD45, 193Ir_DNA, Residual, 103Rh_DNA (life/dead), and 140Ce_bead (Flowjo v. 10.6.1.) Next, the data were compensated in R version 4.1.1 using the CATALYST package and automatic cutoffs. Data were debarcoded with HSNE in Cytosplore. Data were arcsin 5 transformed in R, and batch effects were corrected using the reference samples. The data were downsampled to a maximum of 50,000 cells/sample to both create similar numbers of cells per sample and minimize computational time, while keeping enough cells for in-depth analyzation, and analyzed using the FlowSOM package (22). The downsampled cells were clustered into 100 clusters and gathered in 30 metaclusters for the first overview FlowSOM. Metaclusters with similar phenotypes were then merged, resulting in four groups resembling the major lineages (**Figure 1**; **Supplementary** **Figure S1**). A separate FlowSOM was then performed for each group for in-depth analysis. This two step approach allowed for better in-depth phenotyping as there are less cells in the analysis and small differences can be visualised. For group 1, 2 and 3 a FlowSOM was created with 121 clusters and 100 metaclusters and for group 4, a FlowSOM with 225 clusters and 200 metaclusters was made. Metaclusters with similar phenotypes were merged. Clusters that contained over 500 cells and originated from different samples were included, while doublet clusters were removed (**Supplementary** **Figure S1**). Using the absolute cell counts obtained on fresh blood samples (BD Multitest kit, BD Biosciences) the absolute number of cells in each subset could be calculated. Graphs were generated using Graphpad prism version 8.4.2 by comparing the absolute number of cells at different time points. Any measurements with a value of zero were depicted as a dot on the X-axis. Selected subsets were gated for validation purposes using Flowjo v10.6.1.

### Statistical analysis

For the discovery analysis, the comparisons within one cluster were performed with the Wilcoxon signed rank test in Graphpad prism version 8.4.2 and corrected for multiple testing with Bonferroni.

## Results

### Increase of B cells and CD8^+^ T cells 9 weeks after MSC infusion at week 34

In the Neptune clinical trial allogenic bone marrow-derived MSCs were infused at 25 and 26 weeks post kidney transplantation in 10 patients (**Figure 1**). Notably, no adverse effects directly attributable to the MSC infusions were observed in these patients (age 24-68). All patients maintained a functioning kidney graft at the study’s conclusion, with no occurrences of biopsy-proven acute rejection (BPAR). Detailed clinical information on the study population is available in Dreyer et al. (12).

**Figure 2:**
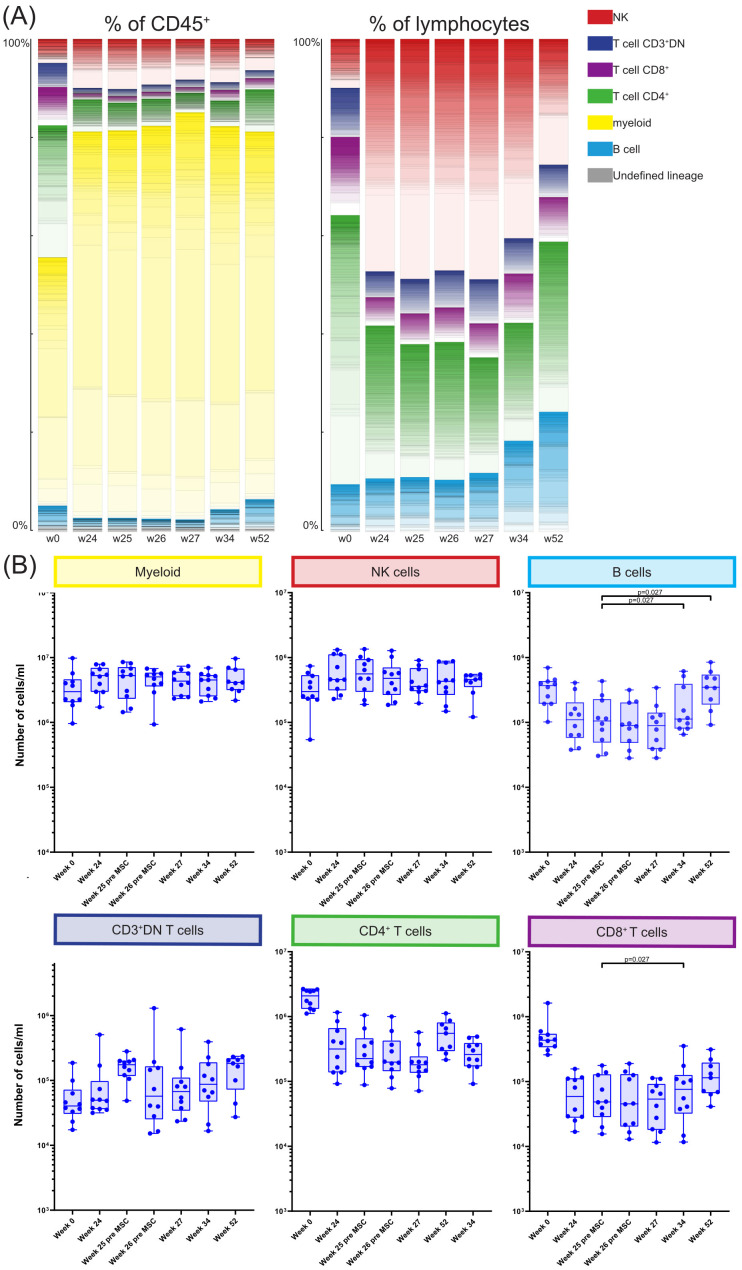
Longitudinal quantification and distribution of major immune cell lineages. **(A)** Graphs showing the number of cells/ml in the MSC treated patients at timepoint w0, w24, w25, w26, w27, w34 and w52, for the six major immune lineages, Myeloid, NK/ILC, B cells, CD3^+^DN Tcells, CD4^+^ T cells and CD8^+^ T cells. Each dot represents an individual patient at the timepoint indicated. P-values were calculated using the Mann-Whitney U test and corrected within each cluster with Bonferroni. **(B)** The contribution of the different cell clusters and major lineages as percentage of CD45^+^ cells (left panel) and as percentage of lymphocytes (right panel), in the MSC treated patients at timepoint w0, w24, w25, w26, w27, w34 and w52.

Participants provided nine blood samples: pre-transplantation (week 0), at weeks 24, 25, and 26 (before and 4 hours after MSC infusion), and at weeks 27, 34, and 52. A 40-marker antibody panel was used with mass cytometry to analyze all samples. Acquired data were analyzed using the FlowSOM clustering method using two steps resulting in 368 phenotypically distinct clusters, as illustrated in **Figure 1** and **Supplementary** **Figure S1**. For each cluster we determined to which major immune lineage it belonged (B cells, myeloid cells, CD3^+^CD4^-^CD8^-^ (CD3^+^DN) T cells, CD4^+^ T cells, CD8^+^ T cells or NK cells, **Supplementary** **Figure S1**). Next, the proportion of each cluster was assessed as percentage of the total CD45^+^ population and as percentage of total lymphocytes at each of the time points (**Figure 2A**). Pre-transplantation (week 0) lymphocytes (49.0%) and myeloid cells (50.4%) each made up half of the total CD45^+^ population. However, at week 24 till week 52 this distribution was skewed towards a dominance of myeloid cells (74.6%-83.4%). Within the lymphocytes, before transplantation (week 0) 54.6% lymphocytes were CD4^+^ T cells, whereas at week 24 till 34 the NK cells made up 40.4%-48.8% of the lymphocytes. While at week 52 the percentage of both B cells and CD4^+^ T cells were again increased, the CD4^+^ T cells remained low compared to week 0 (**Figure 2A**). Due to alemtuzumab induced lymphodepletion these changes in the immune composition were expected and our results confirm that after one year the immune compartment is still not fully recovered.

Next, for each major immune lineage the absolute number of cells at week 25 after transplantation (before MSC infusion) was compared with the number of cells at the following timepoints; week 26 before second infusion of MSC, week 27, week 34 and week 52 (**Figure 2B**). This revealed no differences in the absolute cell numbers between those time points within the myeloid compartment, NK cells, CD3^+^DN T cells and CD4^+^ T cells. However, at week 34 the number of both CD8^+^ T cells and B cells was increased compared to week 25 (both p=0.027). B cell numbers continued to be elevated at week 52 (p=0.027) while the CD8^+^ T cells were not.

### CD3^+^CD4^-^CD8^-^ T cells were decreased 4 hours after first MSC infusion

**Figure 3:**
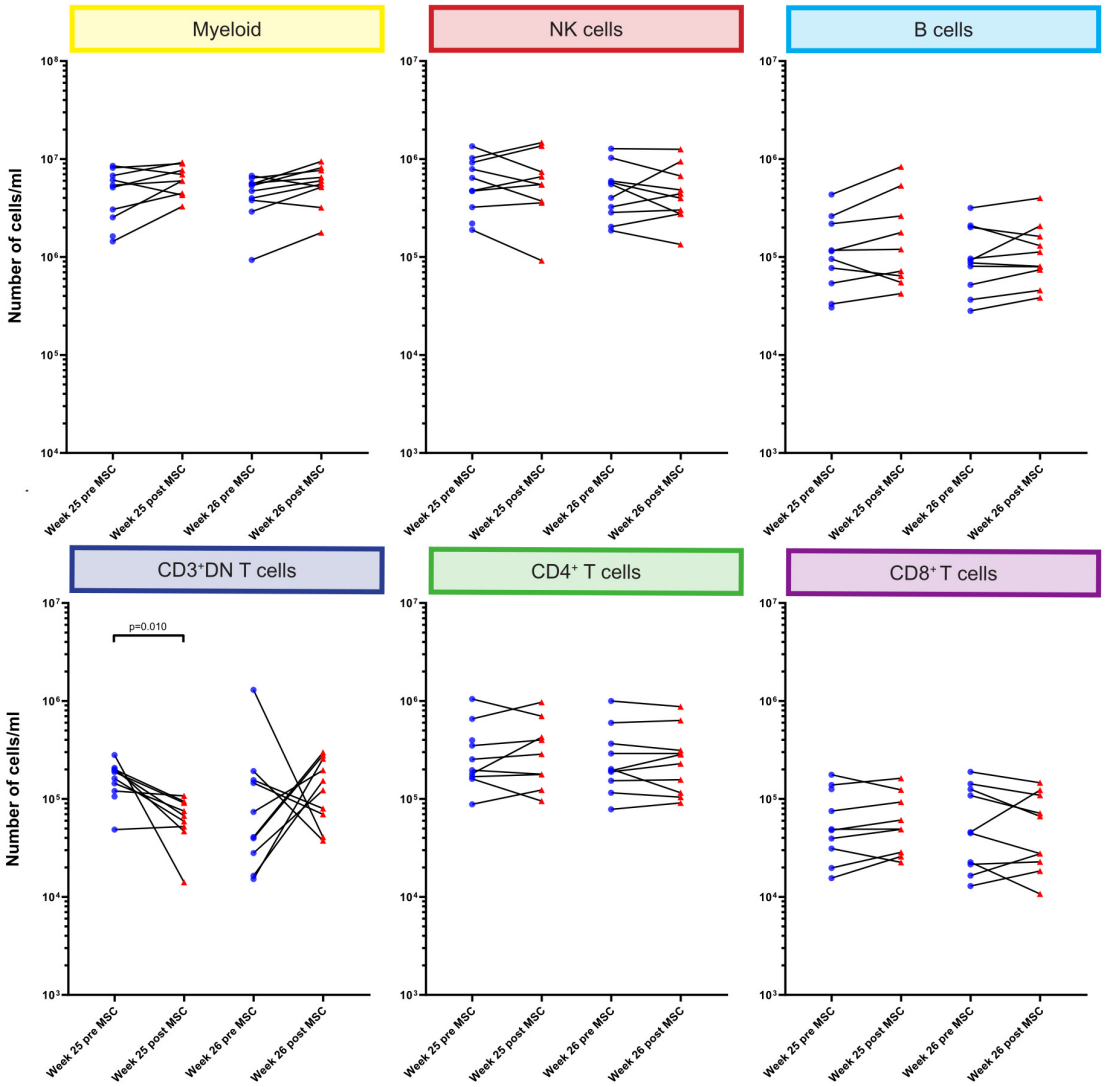
Quantification of major immune cell lineages pre and post MSC therapy. Graphs showing the number of cells/ml in the MSC treated patients at timepoint w25 pre and post MSC and w26 pre and post MSCs for the six major immune lineages, Myeloid, NK/ILC, B cells, CD3^+^DN Tcells, CD4^+^ T cells and CD8^+^ T cells. Each dot represents an individual patient at the timepoint indicated connected with a line to the following timepoint. Blue: pre-MSC infusion, red: 4 hours after MSC infusion. P-values were calculated using the Mann-Whitney U test and corrected within each cluster with Bonferroni.

Although infused MSC are only short lived, we hypothesized that this could still affect circulating immune cells early after MSC infusion. Therefore, we directly compared the absolute numbers of cells at week 25 and week 26 before and 4 hours after each MSC infusion at the major immune lineage level in all individual patients (**Figure 3**). We did not observe significant changes in the major lineages of myeloid cells, NK cells, B cells, CD4^+^ T cells and CD8^+^ T cells upon MSC infusion. However, the number of CD3^+^DN T cells was decreased 4 hours after the first, but not the second, MSC infusion (p=0.010, **Figure 3**).

### Lineage^+^CD11b^+^CD11c^+^CD38^+^CD39^+^Ki-67^+^ subsets are increased 4 hours after each MSC infusion

We next focused on a more detailed analysis of the 368 phenotypically distinct clusters resulting from the FlowSOM clustering. For this we compared the absolute cell numbers of these individual clusters at week 25 and week 26, before and 4 hours after each MSC infusion. This analysis revealed statistically significant differences for 75 clusters (**Supplementary Table S2**). Within these 75 cluster we next combined clusters that exhibited a similar phenotype, resulting in three B cell subsets, seven myeloid subsets, seven CD3^+^DN T cell subsets, four CD8^+^ T cell subsets, 14 CD4^+^ T cell subsets and 14 NK cell subsets, showing a statistically significant increase or decrease when comparing the time points before MSC infusion with the time points 4 hours after MSC infusion (**Supplementary Tables S3-S9**).

Within both the B cells and the T cells we observed several subsets that all shared the expression of CD11b, CD11c, CD38, CD39, and Ki-67 (**Supplementary Tables S3-S9**, indicated in bold). Strikingly, all these subsets were significantly increased 4 hours after MSC infusion, at either one or both infusion moments (**Table 1**). To confirm the changes in these subsets derived from the FlowSOM analysis, we in addition manually gated for these CD11b^+^CD11c^+^CD38^+^CD39^+^Ki-67^+^ phenotypes. Manual gating revealed similarly increased B cell and T cell subsets 4 hours after MSC infusion (**Supplementary** **Figures S2**, **S3A**). Significance was reached for the B cells with this phenotype at week 25, for the CD3^+^DN T cells at both week 25 and week 26, for the CD4^+^ T cells at week 25 and for the CD8^+^ T cells both at week 25 and week 26 (**Supplementary Figures S3B-E**). To conclude, lineage^+^CD11b^+^CD11c^+^CD38^+^CD39^+^Ki-67^+^ subsets discovered in the FlowSOM analysis are increased 4 hours after each MSC infusion which was confirmed with manual gating of these subsets.

**Table 1:** CD11b^+^CD11c^+^CD38^+^CD39^+^Ki-67^+^ B cells and T cells from FLOWSOM analysis.

Lineage	Subset	Cell type	Cells/µl median (range) week 25			Cells/µl median (range) week 26		
			0h	+4h & MSC	p-value	0h	+4h & MSC	-value
**B cells**	2	Proliferating CD11b^+^CD11c^+^CD38^+^CD39^+^ mature B cell	7962 (1767-13262)	14297 (6162-28367)	0.042	5789 (1048-13415)	8351 (2456-16642)	ns
**CD3^+^DN T cells**	1	Proliferating CD11b^+^CD11c^+^CD38^+^CD39^+^ memory T cell	9979 (3506-14865)	14666 (6548-27992)	0.031	5683 (3432-12298)	12023 (4110-15663)	ns
**CD3^+^DN T cells**	3	Proliferating CD11b^+^CD11c^+^CD38^+^CD39^+^CD57^+^ effector T cell	381 (0-693)	848 (176-2095)	0.008	773 (373-2505)	578 (280-2220)	0.012
**CD4+ T cells**	11	Activated proliferating CD11b^+^CD11c^+^CD38^+^CD39^+^Tigit^+^ memory CD4^+^ T cell	1990 (562-4678)	3063 (1417-7482)	0.016	1321 (374-1666)	992 (685-2377)	ns
**CD4+ T cells**	12	Proliferating CD11b^+^CD11c^+^CD38^+^CD39^+^ CD4^+^ T cell	5359 (1729-26545)	10234 (5410-28323)	0.039	3773 (1318-11496)	5312 (1979-15062)	0.008
**CD8+ T cells**	4	Proliferating CD11b^+^CD11c^+^CD38^+^CD39^+^CD27^+^CD127^+^ Tc1-like cytotoxic T cell	2545 (610-7857)	4858 (1708-12136)	ns	1848 (374-3702)	2844 (609-5068)	0.039

ns, not significant.

## Discussion

In the current work we used mass cytometry to gain insight on the impact of MSC therapy on the immune compartment in kidney transplant recipients. In previous work we described the safety and feasibility of the allogenic MSC infusion (12). In the current study we used mass cytometry to visualize the composition of the immune compartment before and up to 52 weeks after kidney transplantation in MSC treated patients. We focused on the effects as soon as 4 hours post MSC transfusion and were able to show significant changes in specific B cell and T cell subsets shortly post MSC transfusion.

It is known that MSCs can impact various immune cell types such as dendritic cells, monocytes, macrophages, B cells, T cells including Treg/Th1/Th2 and Th17 helper cells, NK cells and NKT cells, ILCs, myeloid-derived suppressor cells, neutrophils, and mast cells (6, 7, 13–15). This effect can occur through direct cell-cell contact or indirectly by MSC-derived vesicles, including exosomes and apoptotic bodies, or soluble factors, as reported in studies by Weiss et al. and Jiang et al (23, 24). Upon intravenous infusion MSCs tend to accumulate in the lungs as they cannot pass through narrow capillaries due to their size (17, 25). We have recently shown the rapid death of MCSs upon infusion, determined by MSC-specific cell free DNA measured in plasma 4 hours after infusion (16). In the current study we focused on potential changes in the composition of immune cell subsets at this time point, since massive cell death of MSCs may affect immune cell composition. We showed that absolute cell numbers of three B cell subsets, seven myeloid subsets, seven CD3^+^DN T cell subsets, four CD8^+^ T cell subsets, 14 CD4^+^ T cell subsets (2 Treg), and 14 NK cell subsets were significantly changed 4 hours after infusion compared to pre-infusion. Strikingly, we observed that several of these subsets exhibited an unusual CD11b^+^CD11c^+^CD38^+^CD39^+^Ki-67^+^ phenotype. Manual gating confirmed the increased presence of CD11b/CD11c/CD38/CD39/Ki-67 positivity in B cells, CD3^+^DN, CD4^+^ and CD8^+^ T cells 4 hours after MSC infusion.

CD38, CD39 and Ki-67 are commonly expressed by both B cells and T cells (CD3^+^DN, CD4^+^ and CD8^+^). Both CD38 and CD39 suggest activation and Ki-67 can indicate proliferation (26, 27). CD38, CD39 and Ki-67 could be upregulated by B cells and T cells within the first hours of activation upon encounter with either the apoptotic MSCs or phagocytic cells. Alternatively, increase of these CD38^+^CD39^+^Ki-67^+^ cells could indicate recruitment from adjacent tissues, like the lung, in response to the accumulation of dead MSCs. The integrins CD11b and CD11c are commonly expressed by dendritic cells, monocytes and macrophages. Although not typical, B and T cells can express both CD11b and CD11c (28–30). CD11b^+^CD11c^+^ B cells have been found to strongly stimulate T cells but produce modest levels of secreted antibody (29). While the exact role of CD11c^+^ on T cells is unclear, it has been reported that CD11c may have a regulatory function on CD8^+^ T cells and that these cells have a high migratory capacity (30, 31). While the infusion of MSCs may potentially trigger early and transient upregulation of CD11b and CD11c in B cells and T cells, this remains unexpected and warrants further study. The conduction of a kinetic study with closer intervals could track the marker expression on these cells and thus shed a light on the upregulation of these markers.

The identification of the unusual marker combinations made us consider the formation of doublets. While it is possible that cells stick together and form doublets during staining, doublets were excluded by gating on DNA, width, residual, center and offset excluding the vast majority of doublets. Furthermore, using the barcoding, cells with extra barcodes were also excluded, further mitigating this potential bias. While some cells could theoretically form doublets and contain a single barcode, this is unlikely and would occur equally for all 9 pooled samples of a patient. Therefore these cells would not be elevated specifically 4 hours after each MSC infusion. While limited, doublet formation could still introduce some bias in CyTOF studies, future studies are recommended to use the latest staining techniques to minimize these occurrences and up-to-date post-acquisition data analysis workflow to distinguish these cells.

An alternative explanation for the unusual marker combination can be trogocytosis, a process in which a cell acquires fragments from another living cell. The trogocytic cell has the capability to assimilate membrane proteins from other cells, which can then become integrated in its own plasma membrane (32, 33). While antigen presenting cells (APCs), B cells and T cells all have this ability, the transfer of membrane proteins from APCs to B cells and T cells is best described (34). One could envisage that during the process of trogocytosis, CD11b and CD11c may be transferred from myeloid cells to CD38^+^CD39^+^Ki-67^+^ B cells or T cells, resulting in the CD11b^+^CD11c^+^CD38^+^CD39^+^Ki-67^+^ B cell and T cell phenotype. Trogocytosis of the MSCs, or integration of extracellular vesicles or apoptotic blebs derived from the MSC, are unlikely to explain the observed phenotype as MSCs do not express CD45, CD11b, CD11c, CD38, CD19 or CD3, though they can express CD39 (35). How MSC would drive the increased interaction between myeloid cells and B or T cells, and thereby the process of trogocytosis, is currently unknown. In our previous work we tracked the response of 29 cytokines 4 hours after each MSC infusion (12). TNFα showed a significant decrease 4 hours after the first MSC infusion however this effect was not seen after the second infusion. Anti-inflammatory cytokine IL4 showed small though significant differences after both the first and second infusion. Anti-inflammatory cytokine IL10 was decreased after each infusion, which was significant after the second infusion. Proinflammatory cytokine IFNγ showed non-significant decrease at both time points. No major changes were observed 4 hours after MSC infusions for the other cytokines. The responses of these cytokines, anti-inflammatory as well as proinflammatory, are systemic in nature, and therefore a direct association with the identified cells cannot be definitively established.

In previous work we showed that while absolute numbers of monocytes, B cells, NK cells and CD8^+^ T cells remained stable in the two weeks after infusion (week 26 and 27), CD4^+^ T cells increased in the second week post MSC infusion (week 27). However, this could be due to the immune cell repopulation as a consequence of induction therapy with alemtuzumab (12). In the current study we could confirm that B cells, CD4^+^ T cells and CD8^+^ T cells did not reach their base line levels at week 25, the time of the first MSC infusion (12). Repopulation after induction therapy was still ongoing at 52 weeks, as the number of CD4^+^ T cells and CD8^+^ T cells was still lower compared to baseline. Unexpectedly, our data show that the absolute number of CD3^+^DN T cells was not decreased at 24 weeks compared to baseline. This double negative population was not studied in our previous report. These data indicate that either the CD3^+^DN T cells are less efficiently depleted by the induction therapy or they repopulate quicker within 24 weeks compared to both CD4^+^ and CD8^+^ T cells.

The transient upregulation of markers such as CD11b, CD11c, CD38, CD39, and Ki-67 on B cells and T cells shortly after MSC infusion suggests early immune activation. However, it remains to be established whether this contributes to potential long-term beneficial clinical effects. Autologous MSC infusion has been exploited for a safe reduction in immunosuppressive drugs (9), and has been proposed to induce an immune regulatory milieu (10). Both CD38 and CD39 play key roles in the adenosine pathway, which is known to regulate immune responses by generating the immunosuppressive molecule adenosine (36, 37). The extracellular adenosine level normally kept low under physiological conditions, but it increases during inflammation and cell death which could be triggered by the massive cell death of MSCs (37). CD38, through its enzymatic activity, influences the metabolism of NAD^+^, indirectly contributing to the generation of substrates such as ATP and ADP, which are crucial for the adenosine-producing activity of CD39. CD39 further catalyses the conversion of ATP and ADP into AMP, a precursor of adenosine. Unfortunately, we do not have information on CD73, a molecule required for the final conversion of AMP to adenosine. Future studies should incorporate CD73 to provide a more comprehensive view of adenosine regulation and its potential role in immune modulation via this pathway. This could reveal whether the transient increase in CD11b^+^CD11c^+^CD38^+^CD39^+^Ki-67^+^ B cells and T cells contributes to a sustained immunosuppressive or regulatory environment, particularly through the adenosine pathway, and help clarify the longer-term effects of MSCs infusion on allograft survival in kidney transplantation.

The current research involved an unbiased discovery analysis of various immune cell markers, leading to the identification of numerous distinct clusters/subsets. As a result, a large number of comparisons were made. In this study we corrected with Bonferroni to account for multiple comparisons within a single cluster. Correcting for false positives across the entire study would require extremely low p-values to remain significant after correction. Therefore, we argue that the subsets discovered in this study should be validated and examined more closely in future studies to determine their potential role in MSC therapy. As the current study was a single center study with a limited number of patients, these future studies are recommended to involve multiple centers and include a larger number of patients.

In conclusion, we here report an extensive description of the immune cell composition in kidney transplantation patients 4 hours after receiving MSC therapy. While the mechanisms of action are still unclear, our results indicate that subsets of cells within all the immune cell lineages respond to MSC infusion as soon as 4 hours. We highlight the discovery of CD11b^+^CD11c^+^CD38^+^CD39^+^Ki-67^+^ B and T cell subsets which increased consistently 4 hours after MSC infusion. Our findings may facilitate in the ongoing quest to understand the effect of MSC therapy on the immune system.

## Data Availability

The raw data supporting the conclusions of this article will be made available by the authors, without undue reservation.
