# Supplementary material for: Allogenic MSC infusion in kidney transplantation recipients promotes within 4 hours distinct B cell and T cell phenotypes
Source: Front Immunol. 2024 Oct 9;15:1455300. doi: 10.3389/fimmu.2024.1455300 (PMC11500071; doi:10.3389/fimmu.2024.1455300)
Supplement: Supplementary file 1 [file DataSheet1.zip › Figure S3.PDF]

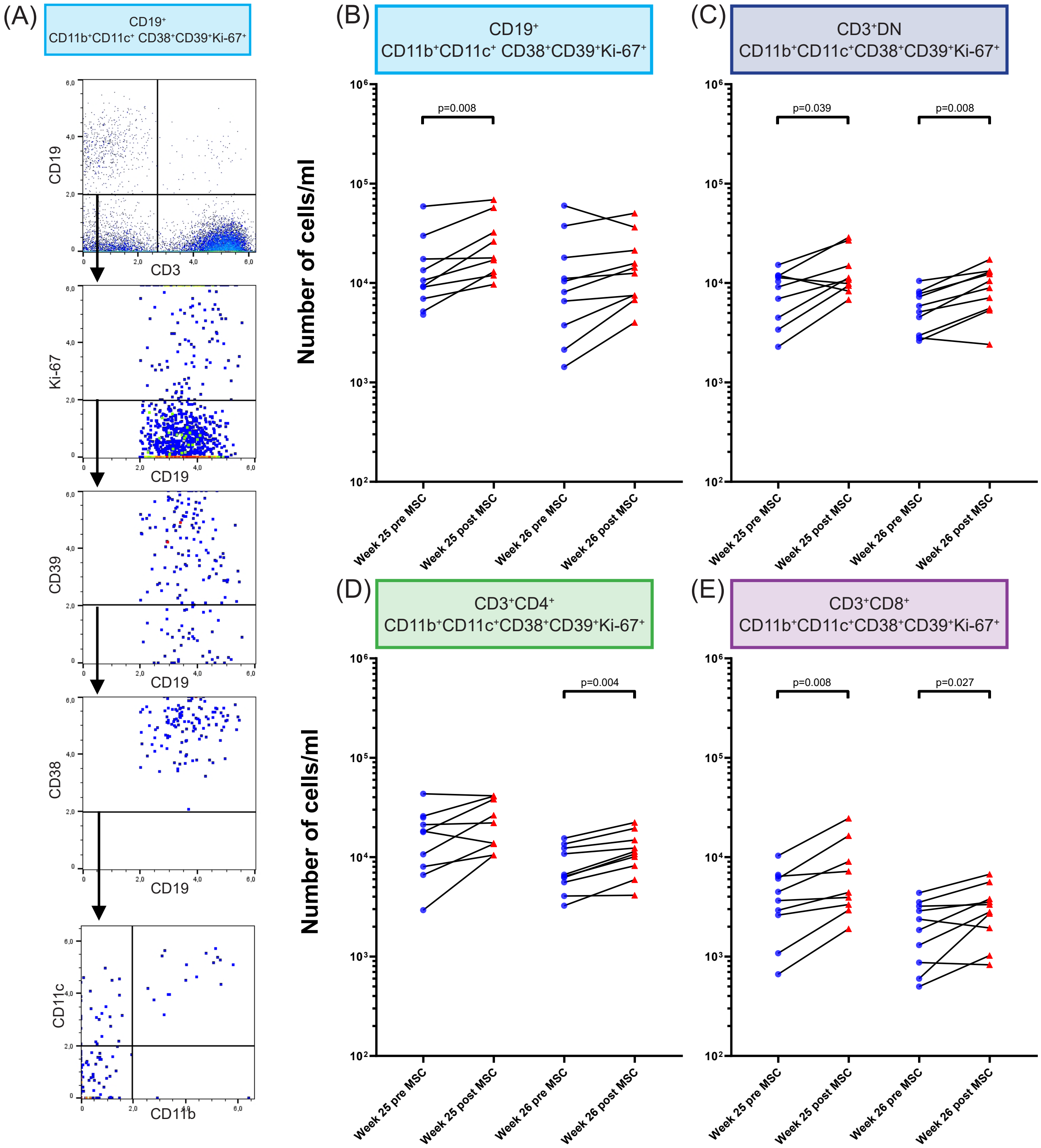

Figure S3. **Manual gating of CD11b⁺CD11c⁺CD38⁺CD39⁺Ki-67⁺ B cells and T cells.** Graphs showing the number of cells/ml in the MSC treated patients at w25 and w26 before and after MSC infusion. **A.** Representation of gating strategy. **B.** $CD19^+CD11b^+CD11c^+CD38^+CD39^+Ki\text{-}67^+$. **C.** $CD3^+CD4^-CD8^-CD11b^+CD11c^+$ $CD38^+CD39^+Ki\text{-}67^+$. **D.** $CD3^+CD4^+CD11b^+CD11c^+CD38^+CD39^+Ki\text{-}67^+$. E. $CD3^+CD8^+CD11b^+CD11c^+$ $CD38^+CD39^+Ki\text{-}67^+$. Red: 4 hours after MSC infusion, blue: pre-MSC infusion. P-values were calculated with the Mann-Whitney U test and corrected within each cluster with Bonferroni.
