# Supplementary material for: Allogenic MSC infusion in kidney transplantation recipients promotes within 4 hours distinct B cell and T cell phenotypes
Source: Front Immunol. 2024 Oct 9;15:1455300. doi: 10.3389/fimmu.2024.1455300 (PMC11500071; doi:10.3389/fimmu.2024.1455300)
Supplement: Supplementary file 1 [file DataSheet1.zip › Supplementary table S1-S9.DOCX]

**­­Supplementary Tables**

**Table S1.**

|  | Antigen | Tag | Clone | Company | Cat# | Lot# | Dilution |
| --- | --- | --- | --- | --- | --- | --- | --- |
| 1 | CD45 | 89y | HI30 | FLM | 3089003B | 3321821 | 1:100 |
| 2 | HLA-DR | 115ln | L243 | BioL | 307651 | B262205 | 1:100 |
| 3 | CCR6 (CD196 | 141 Pr | G034E3 | FLM | 3141003A | 0671821 and 3471805 | 1:100 |
| 4 | CD19 | 142 Nd | HIB19 | FLM | 3142001B | 171815 | 1:200 |
| 5 | CD5 | 143 Nd | UCHT2 | FLM | 3143007B | 451808 | 1:200 |
| 6 | CD69 | 144 Nd | FN50 | FLM | 3144018B | 3041705 | 1:200 |
| 7 | CD4 | 145 Nd | RPA-T4 | FLM | 3145001B | 2001809 | 1:100 |
| 8 | CD8 | 146 Nd | RPAT8 | FLM | 3146001B | 1201824 | 1:100 |
| 9 | CD40 | 147 Sm | 5C3 | BioLegend | 334325 | B254704 | 1:200 |
| 10 | PD-L1 | 148 Nd | 29E.2A3 | FLM | 3148017B | 371816 | 1:200 |
| 11 | CD25 (IL2-R) | 149 Sm | 2A3 | FLM | 3149010B | 1351815 | 1:100 |
| 12 | CD57 | 150 Nd | HNK-1 | BioLegend | 359602 | B256178 | 1:200 |
| 13 | FoxP3 | 151 Eu | 259D/C7 | BD | 560044 | 8172636 | 1:100 |
| 14 | TCRgd | 152 Sm | 11F2 | FLM | 3152008B | 441915 | 1:50 |
| 15 | CD7 | 153 Eu | CD7-6B7 | FLM | 3153014B | 3181407 | 1:100 |
| 16 | Tigit | 154 Sm | ML5 | BioLegend | 311127 | 1501812 | 1:100 |
| 17 | CD27 | 155 Gd | L128 | FLM | 3155001B | 3181710 | 1:200 |
| 18 | CXCR3 | 156 Gd | G025H7 | FLM | 3156004B | 2001807 | 1:100 |
| 19 | CD22 | 157 Gd | HIB22 | BioLegend | 302511 | B214802 | 1:400 |
| 20 | CD3 | 158 Gd | UCHT1 | BioLegend | 300443 | B242212 | 1:400 |
| 21 | CCR7 (CD197) | 159 Tb | G043H7 | FLM | 3159003A | 1691906 | 1:100 |
| 22 | Tbet | 160 Gd | 4B10 | FLM | 3160010B | 671822 | 1:50 |
| 23 | KLRG-1 | 161 Dy | REA261 | MACS | 120-014-229 | 5190307174 | 1:100 |
| 24 | CD11c | 162 Dy | Bu15 | FLM | 3162005B | 1671707 | 1:200 |
| 25 | CD39 | 163 Dy | A1 | BioLegend | 328221 | B242203 | 1:200 |
| 26 | CD161 | 164 Dy | HP-3G10 | FLM | 3164009B | 451815 | 1:100 |
| 27 | CD127 (IL-7Ra) | 165 Ho | A019D5 | FLM | 3165008B | 2541802 | 1:100 |
| 28 | IgM | 166 Er | MHM-88 | BioLegend | 314527 | B208787 | 1:400 |
| 29 | GATA-3 | 167 Er | TWAJ | FLM | 3167007A0 | 391808 | 1:100 |
| 30 | Ki-67 | 168 Er | B56 | FLM | 3168007B | 581816 | 1:50 |
| 31 | EOMES | 169 Tm | WD1928 | eBioscience | 14-4877-82 | 1944573 | 1:300 |
| 32 | CTLA-4 (CD152) | 170 Er | 14D3 | FLM | 317005B | 711815 | 1:100 |
| 33 | CD40L (CD154) | 171 Yb | 24-31 | BioLegend | 310835 | B232943 | 1:200 |
| 34 | CD38 | 172 Yb | HIT2 | FLM | 3172007B | 941814 | 1:200 |
| 35 | CD45RO | 173 Yb | UCHL1 | BioL | 304239 | B256595 | 1:100 |
| 36 | CD21 | 174 Yb | B-ly-4 | BD | 555421 | 7326840 | 1:200 |
| 37 | PD-1 | 175 Lu | EH 12.2H7 | FLM | 3175008B | 371819 | 1:100 |
| 38 | CD56 | 176 Yb | NCAM16.2 | FLM | 3176008B | 1671712 | 1:100 |
| 39 | CD45RA | 198 Pt | HI100 | BioLegend | 304143 | B186475 | 1:200 |
| 40 | CD11b | 209Bi | ICRF44 | FLM | 3209003B | 3091802 | 1:100 |

**Table S2.**

| Subset | Cluster | Expression | Cell type |
| --- | --- | --- | --- |
|  | **B cells** |  |  |
| 1 | 181 | CD19+IgM+CD11b+CD127+CCR6+CD25+ | IgM+CD11b+CD127+CCR6+CD25+ B cell |
| 2 | 193 | CD45RA+CD45RO+HLA-DR+CD19+CD21+CD22+IgM+CD11b+CD11c+CD38+CD39+Ki-67+ | **Proliferating CD11b+CD11c+CD38+CD39+ mature B cell** |
|  | 194 | CD45RA+CD45RO+HLA-DR+CD19+CD21+CD22+IgM+CD11b+CD11c+CD38+CD39+CCR7+Ki-67+ |  |
|  | 195 | CD45RA+HLA-DR+CD19+CD21+CD22+IgM+CD11b+CD11c+CD38+CD39+Ki-67+ |  |
| 3 | 238 | CD45RA+HLA-DR+CD19+CD21+CD22+IgM+CD69+CD40L+ | CD69+CD40L+ mature B cell |
|  |  |  |  |
|  | **Myeloid** |  |  |
| 1 | 250 | CD45RO+HLA+DR+IgM+CD11b+CD11c+CD38+CD39+Ki+67+ | Proliferating IgM+CD11b+ CD11c+CD38+CD39+ monocyte/macrophage |
| 2 | 256 | CD45RA+CD45RO+HLA+DR+CD11b+CD11c+CD38+CD39+Ki+67+ | Proliferating CD11b+CD11c+CD38+CD39+ monocyte/macrophage |
|  | 257 | CD45RO+HLA-DR+CD11b+CD11c+CD38+CD39+CD40L+Ki-67+ |  |
|  | 258 | CD45RO+HLA-DR+CD11b+CD11c+CD38+CD39+Ki-67+ |  |
|  | 260 | CD45RO+CD11b+CD11c+CD38+CD39+CD40L+Ki-67+ |  |
|  | 266 | CD45RO+HLA-DR+CD11b+CD11c+CD38+CD39+CXCR3+Ki-67+ |  |
|  | 269 | HLA-DR+CD11b+CD11c+CD38+CD39+Ki-67+ |  |
| 3 | 344 | CD45RO+CD11b+CD11c+CD38+CD39+CD57+Tbet+Ki+67+ | Proliferating CD11b+CD11c+CD38+CD39+Tbet+ monocyte/macrophage |
|  | 347 | CD45RO+CD11b+CD11c+CD38+CD39+EOMES+Tbet+Ki+67+ |  |
| 4 | 255 | CD45RO+CD11b+CD11c+CD38+CD39+ | CD11b+CD11c+CD38+CD39+ monocyte/macrophage |
| 5 | 277 | HLA-DR+CD11b+CD11c+CD39+CXCR3+ | CD11b+CD11c+CD39+ CXCR3+ monocyte/macrophage |
| 6 | 282 | CD45RO+CD11b+CD38+CD40L+ | CD11b+ monocyte/macrophage |
| 7 | 274 | CD45RA+HLA+DR+CD11c+CD39+ | CD11c+ monocyte/macrophage |
|  | 275 | CD45RA+CD11c+ |  |
|  |  |  |  |
|  | **CD3+CD4-CD8-** | |  |
| 1 | 173 | CD45RO+CD3+CD7+CD11b+CD11c+CD27+CD38+CD39+CD127+EOMES+Tbet+Ki-67+ | **Proliferating CD11b+CD11c+CD38+CD39+ memory T cell** |
|  | 174 | CD45RO+HLA-DR+CD3+CD7+CD11b+CD11c+CD27+CD38+CD39+CD127+Ki-67+ |  |
| 2 | 175 | CD45RO+CD3+CD7+CD11b+CD11c+CD27+CD127+Ki-67+ | Proliferating CD11b+CD11c+ memory T cell |
| 3 | 341 | CD45RA+CD45RO+CD3+CD7+CD11b+CD11c+CD38+CD39+CD57+KLRG1+Tbet+Ki+67+ | **Proliferating CD11b+CD11c+CD38+CD39+CD57+ effector T cell** |
| 4 | 59 | CD3+ | T cell |
| 5 | 64 | CD45RO+CD3+CD5+CD27+CD69+CD127+CD40L+ | Activated CD40L+ T cell |
|  | 152 | CD3+CD5+CD7+CD27+CD69+CD127+CTLA-4+CD40L+ |  |
| 6 | 67 | CD45RO+CD3+CD5+CD7+CD27+CD127+ | Memory T cell |
| 7 | 162 | CD45RO+CD3+CD7+CD127+CD161+KLRG1+EOMES+ | CD161+ central memory T cell |
|  |  |  |  |
|  | **CD4+ cells** |  |  |
| 1 | 168 | CD45RA+CD3+CD4+CD5+CD7+CD11c+CD27+CD127+CCR7+ | CD11c+CD7+CD27+CD127+ naïve CD4+ T cell |
| 2 | 103 | CD45RO+CD3+CD4+CD5+CD27+CD127+CCR7+ | Central memory CD4+ T cell |
| 3 | 1 | CD45RA+CD45RO+CD3+CD4+CD5+CD7+CD56+CD27+CD57+CD127+EOMES+Tbet+CD40L+ | CD56+ Th-1 like cell |
|  | 171 | CD45RA+CD3+CD4+CD5+CD7+CD56+CD27+CD127+CCR7+EOMES+Tbet+ |  |
| 4 | 59 | CD45RO+CD3+CD4+CD5+CD11b+CD127+CD40L+ | CD11b+ memory CD4+ T cell |
|  | 82 | CD45RO+CD3+CD4+CD5+CD11b+CD27+CD127+ |  |
| 5 | 111 | CD45RO+CD3+CD4+CD5+CD7+CD27+CD127+CXCR3+CD40L+ | CXCR3+CD40L+ memory CD4+ T cell |
| 6 | 77 | CD45RO+CD3+CD4+CD5+CD27+CD127+CD161+CD40L+ | CD161+ memory CD4+ T cell |
|  | 95 | CD45RO+CD3+CD4+CD5+CD7+CD27+CD127+CD161+ |  |
| 7 | 102 | CD45RO+CD3+CD4+CD5+CD7+CD27+CD127+CD161+CCR7+PD+1+Tigit+CD40L+ | CD161+PD-1+Tigit+ memory CD4+ T cell |
| 8 | 109 | CD45RO+CD3+CD4+CD5+CD7+CD27+CD127+PD+1+CD40L+ | PD-1+ memory CD4+ T cell |
| 9 | 117 | CD3+CD4+CD5+CD7+CD27+CD127+CCR7+CD25+ | Activated naive CD4+ T cell |
|  | 118 | CD45RA+CD3+CD4+CD5+CD7+CD27+CD127+CCR7+CD25+ |  |
| 10 | 158 | CD45RO+CD3+CD4+CD5+CD7+CD27+CD38+CD25+CTLA-4+Ki+67+ | Activated proliferating CD38+CTLA-4+ memory CD4+ T cell |
| 11 | 176 | CD45RO+CD3+CD4+CD5+CD7+CD11b+CD11c+CD27+CD38+CD39+CD25+Tigit+Ki-67+ | **Activated proliferating CD11b+CD11c+CD38+CD39+Tigit+ memory CD4+ T cell** |
| 12 | 178 | CD45RA+CD45RO+CD3+CD4+CD5+CD7+CD11b+CD11c+CD27+CD38+CD39+CD127+CCR7+Ki-67+ | **Proliferating CD11b+CD11c+CD38+CD39+ CD4+ T cell** |
|  |  |  |  |
|  | **Treg** |  |  |
|  | 44 | CD45RO+CD3+CD4+CD5+CD7+CD11b+CD27+CD39+CD127+CD25+Tigit+FoxP3+ | CD7+CD11b+CD127+Tigit+ Treg |
|  | 45 | CD45RO+CD3+CD4+CD27+CD39+CD25+FoxP3+ | Treg |
|  | 46 | CD45RO+CD3+CD4+CD5+CD7+CD39+CD25+Tigit+FoxP3+ | CD7+Treg |
|  | 47 | CD45RO+CD3+CD4+CD5+CD27+CD39+CD25+Tigit+FoxP3+ | Tigit+ Treg |
|  | 48 | CD45RO+CD3+CD4+CD5+CD7+CD27+CD39+CD161+PD-1+CD25+Tigit+FoxP3+ | CD161+PD-1+Tigit+ Treg |
|  |  |  |  |
|  | **CD8+ cells** |  |  |
| 1 | 41 | CD45RA+CD3+CD8+CD5+CD7+CD57+KLRG1+EOMES+Tbet+ | CD57+ Tc1-like naïve cytotoxic T cell |
|  | 175 | CD45RA+CD3+CD8+CD5+CD57+KLRG1+Tbet+ |  |
| 2 | 128 | CD45RA+CD3+CD8+CD5+CD7+CD27+CD127+CXCR3+CCR7+ | CD27+CD127+CXCR3+ Tc1-like naïve cytotoxic T cell |
| 3 | 142 | CD45RO+CD3+CD8+CD5+CD7+CD27+CD127+KLRG1+Tigit+EOMES+ | CD27+CD127+Tigit+ Tc1-like memory cytotoxic T cell |
| 4 | 172 | CD45RA+CD45RO+CD3+CD8+CD5+CD7+CD11b+CD11c+CD27+CD38+CD39+CD127+CCR7+Ki-67+ | **Proliferating CD11b+CD11c+CD38+CD39+CD27+CD127+ Tc1-like cytotoxic T cell** |
|  |  |  |  |
|  | **NK cells** |  |  |
| 1 | 316 | CD45RA+CD56+EOMES+Tbet+ | NK cell |
|  | 330 | CD45RA+CD7+CD56+CD27+EOMES+Tbet+ |  |
|  | 331 | CD45RA+CD7+CD56+KLRG1+EOMES+Tbet+ |  |
|  | 336 | CD45RA+CD7+CD56+EOMES+Tbet+ |  |
| 2 | 340 | CD45RA+CD7+CD56+KLRG1+Tigit+EOMES+Tbet+ | Tigit+ NK cell |
| 3 | 337 | CD45RA+CD7+CD56+CD38+EOMES+Tbet+ | CD38+ NK cell |
| 4 | 328 | CD45RA+CD7+CD56+CD38+CD39+EOMES+Tbet+ | CD38+CD39+ NK cell |
| 5 | 326 | CD45RA+CD7+CD56+CD38+CD39+CD161+EOMES+Tbet+ | CD38+CD39+CD161+ NK cell |
| 6 | 311 | CD7+CD56+CD11c+EOMES+Tbet+ | CD11c+ NK cell |
|  | 315 | CD45RA+CD56+CD11c+EOMES+Tbet+ |  |
| 7 | 312 | CD45RA+CD7+CD56+CD11c+CD27+CD38+EOMES+Tbet+ | CD11c+CD38+NK cell |
|  | 313 | CD45RA+CD7+CD56+CD11c+CD27+CD38+CD127+EOMES+Tbet+ |  |
| 8 | 305 | CD45RA+CD7+CD56+CD11c+CD38+CD39+EOMES+Tbet+ | CD11c+CD38+CD39+ NK cell |
|  | 309 | CD7+CD56+CD11c+CD27+CD38+CD39+EOMES+Tbet+ |  |
|  | 310 | CD45RA+CD7+CD56+CD11c+CD27+CD38+CD39+CD127+EOMES+Tbet+ |  |
| 9 | 303 | CD45RA+CD7+CD56+CD11c+CD127+CD161+EOMES+Tbet+ | CD11c+CD161+ NK cell |
| 10 | 342 | CD45RA+CD7+CD56+CD57+CD127+EOMES+Tbet+ | CD57+ NK cell |
| 11 | 345 | CD45RA+CD45RO+CD7+CD56+CD11b+CD11c+CD38+CD39+CD57+EOMES+Tbet+Ki-67+ | Proliferating CD11b+CD11c+CD38+CD39+CD57+ NK cell |
| 12 | 348 | CD45RA+CD45RO+HLA-DR+CD7+CD56+CD11b+CD11c+CD38+CD39+EOMES+Tbet+Ki-67+ | Proliferating CD11b+CD11c+CD38+CD39+ NK cell |
|  | 350 | CD45RO+HLA-DR+CD7+CD56+CD11b+CD11c+CD38+CD39+EOMES+Tbet+Ki-67+ |  |
|  | 351 | CD45RO+CD7+CD56+CD11b+CD11c+CD38+CD39+EOMES+Tbet+Ki-67+ |  |
|  | 354 | CD45RA+CD45RO+CD7+CD56+CD11b+CD11c+CD38+CD39+EOMES+Tbet+Ki-67+ |  |
| 13 | 359 | CD45RA+CD7+CD56+CD11c+CD38+CD39+EOMES+Tbet+Ki+67+ | Proliferating CD11c+CD38+CD39+ NK cell |
| 14 | 362 | CD7+CD56+CD11c+CD27+CD38+EOMES+Tbet+Ki-67+ | Proliferating CD11c+CD38+ NK cell |

**­­­**

**Table S3. B cells**

| Subset | Cell type | Cells/µl  median (range)  week 25 | |  | Cells/µl  median (range)  week 26 | |  |
| --- | --- | --- | --- | --- | --- | --- | --- |
|  |  | **0h** | **+4h & MSC** | **p-value** | **0h** | **+4h & MSC** | **p-value** |
| 1 | IgM+CD11b+CD127+CCR6+CD25+ B cell | 208  (0-22178) | 810  (0-57797) | ns | 211  (0-5498) | 496  (0-8709) | 0.004 |
| 2 | **Proliferating CD11b+CD11c+CD38+CD39+ mature B cell** | **7962**  **(1767-13262)** | **14297**  **(6162-28367)** | **0.042** | **5789**  **(1048-13415)** | **8351**  **(2456-16642)** | **ns** |
| 3 | CD69+CD40L+ mature B cell | 786  (229-1455) | 636  (0-3224) | 0.008 | 0  (0-563) | 642  (169-2783) | 0.012 |

**Table S4. Myeloid cells**

| Subset | Cell type | Cells/µl  median (range)  week 25 | |  | Cells/µl  median (range)  week 26 | |  |
| --- | --- | --- | --- | --- | --- | --- | --- |
|  |  | **0h** | **+4h & MSC** | **p-value** | **0h** | **+4h & MSC** | **p-value** |
| 1 | Proliferating IgM+CD11b+ CD11c+CD38+CD39+ monocyte/macrophage | 5245  (2286-51180) | 9396  (5810-34079) | ns | 5232  (1529-17493) | 10079  (4047-39392) | 0.020 |
| 2 | Proliferating CD11b+CD11c+CD38+CD39+ monocyte/macrophage | 1695455  (639277-3289937) | 2636824  (1381715-4569993) | 0.016 | 1411312  (445589-2799903) | 2392043  (1053523-3929906) | 0.004 |
| 3 | Proliferating CD11b+CD11c+CD38+CD39+Tbet+ monocyte/macrophage | 4158  (0-9132) | 6073  (898-10592) | 0.023 | 3637  (0-5498) | 4991  (699-7569) | 0.039 |
| 4 | CD11b+CD11c+CD38+CD39+ monocyte/macrophage | 67367  (11813-389607) | 83126  (24391-426109) | ns | 56514  (9653-333305) | 86217  (13187-477878) | 0.039 |
| 5 | CD11b+CD11c+CD39+ CXCR3+ monocyte/macrophage | 14934  (3658-81891) | 34351  (12136-86795) | 0.016 | 15347  (3888-91788) | 25590  (783-317437) | ns |
| 6 | CD11b+ monocyte/macrophage | 1415  (369-6583) | 4247  (451-17730) | ns | 1897  (624-6831) | 4396  (473-14115) | 0.039 |
| 7 | CD11c+ monocyte/macrophage | 42828  (9222-151730) | 17961  (10619-48761) | 0.023 | 56223  (4018-167769) | 22892  (1436-111418) | ns |

**Table S5. CD3+CD4-CD8- T cells**

| Subset | Cell type | Cells/µl  median (range)  week 25 | |  | Cells/µl  median (range)  week 26 | |  |
| --- | --- | --- | --- | --- | --- | --- | --- |
|  |  | **0h** | **+4h & MSC** | **p-value** | **0h** | **+4h & MSC** | **p-value** |
| 1 | **Proliferating CD11b+CD11c+CD38+CD39+ memory T cell** | **9979**  **(3506-14865)** | **14666**  **(6548-27992)** | **0.031** | **5683**  **(3432-12298)** | **12023**  **(4110-15663)** | **ns** |
| 2 | Proliferating CD11b+CD11c+ memory T cell | 3448  (728-5245) | 6478  (2394-9180) | ns | 2843  (1274-6333) | 4139  (1446-7508) | 0.004 |
| 3 | **Proliferating CD11b+CD11c+CD38+CD39+CD57+ effector T cell** | **381**  **(0-693)** | **848**  **(176-2095)** | **0.008** | **773**  **(373-2505)** | **578**  **(280-2220)** | **0.012** |
| 4 | T cell | 1311  (629-4420) | 1820  (424-8541) | ns | 1134  (124-10746) | 2361  (415-10967) | 0.012 |
| 5 | Activated CD40L+ T cell | 2978  (739-8573) | 6869  (2843-18421) | 0.023 | 197  (0-1333) | 5251  (914-7272) | 0.004 |
| 6 | Memory T cell | 1677  (562-4134) | 2120  (743-5410) | ns | 1147  (145-10614) | 1596  (338-12926) | 0.008 |
| 7 | CD161+ central memory T cell | 1886  (425-3817) | 2843  (1114-7433) | ns | 1160  (435-3461) | 2434  (676-8109) | 0.012 |

| Subset | Cell type | Cells/µl  median (range)  week 25 | |  | Cells/µl  median (range)  week 26 | |  |
| --- | --- | --- | --- | --- | --- | --- | --- |
|  |  | **0h** | **+4h & MSC** | **p-value** | **0h** | **+4h & MSC** | **p-value** |
| 1 | CD11c+CD7+CD27+CD127+ naïve CD4+ T cell | **991**  **(312-2336)** | 2558  (1021-7138) | 0.016 | **1096**  **(132-2166)** | 2750  (491-3476) | 0.004 |
| 2 | Central memory CD4+ T cell | 425  (0-1886) | 1151  (150-4789) | 0.039 | 696  (167-2637) | 1041  (457-3929) | ns |
| 3 | CD56+ Th-1 like cell | 1801  (562-8115) | 3944  (557-12812) | 0.016 | 1651  (248-6471) | 2260  (145-7727) | ns |
| 4 | CD11b+ memory CD4+ T cell | 3185  (970-14851) | 5668  (464-32457) | 0.023 | 1612  (393-11840) | 3430  (914-13719) | 0.027 |
| 5 | CXCR3+CD40L+ memory CD4+ T cell | 369  (0-1072) | 528  (212-5125) | 0.023 | 399  (0-17603) | 508  (127-7442) | ns |
| 6 | CD161+ memory CD4+ T cell | 3144  (375-6445) | 3605  (1114-3605) | 0.016 | 1759  (0-9129) | 2562  (507-10967) | ns |
| 7 | CD161+PD-1+Tigit+ memory CD4+ T cell | 3740  (187-17529) | 2533  (810-14236) | ns | 2994  (580-6064) | 2171  (338-4056) | 0.020 |
| 8 | PD-1+ memory CD4+ T cell | 1351  (157-5183) | 1056  (0-8826) | ns | 1765  (0-17079 | 681  (140-12273) | 0.020 |
| 9 | Activated naive CD4+ T cell | 40937  (19480-138403) | 37743  (17079-155454) | ns | 43032  (25389-80722) | 31855  (20722-74552) | 0.039 |
| 10 | Activated proliferating CD38+CTLA-4+ memory CD4+ T cell | 381  (0-693) | 848  (176-2095) | 0.023 | 773  (373-2505) | 578  (280-2220) | ns |
| 11 | **Activated proliferating CD11b+CD11c+CD38+CD39+Tigit+ memory CD4+ T cell** | **1990**  **(562-4678)** | **3063**  **(1417-7482)** | **0.016** | **1321**  **(374-1666)** | **992**  **(685-2377)** | **ns** |
| 12 | **Proliferating CD11b+CD11c+CD38+CD39+ CD4+ T cell** | **5359**  **(1729-26545)** | **10234**  **(5410-28323)** | **0.039** | **3773**  **(1318-11496)** | **5312**  **(1979-15062)** | **0.008** |

**Table S6. CD4+ T cells**

| Subset | Cell type | Cells/µl  median (range)  week 25 | |  | Cells/µl  median (range)  week 26 | |  |
| --- | --- | --- | --- | --- | --- | --- | --- |
|  |  | **0h** | **+4h & MSC** | **p-value** | **0h** | **+4h & MSC** | **p-value** |
| 1 | CD57+ Tc1-like naïve cytotoxic T cell | 1607  (0-17984) | 835  (0-17358) | ns | 387  (0-21357) | 101  (0-11624) | 0.016 |
| 2 | CD27+CD127+CXCR3+ Tc1-like naïve cytotoxic T cell | 1296  (369-3740) | 854  (0-5158) | ns | 1740  (499-26689) | 1599  (76-14362) | 0.012 |
| 3 | CD27+CD127+Tigit+ Tc1-like memory cytotoxic T cell | 849  (0-3062) | 449  (0-1949) | ns | 754  (145-4596) | 498  (0-1678) | 0.020 |
| 4 | **Proliferating CD11b+CD11c+CD38+CD39+CD27+CD127+ Tc1-like cytotoxic T cell** | **2545**  **(610-7857)** | **4858**  **(1708-12136)** | **ns** | **1848**  **(374-3702)** | **2844**  **(609-5068)** | **0.039** |

**Table S7. CD8+ T cells**

| Cluster | Cell type | Cells/µl  median (range)  week 25 | |  | Cells/µl  median (range)  week 26 | |  |
| --- | --- | --- | --- | --- | --- | --- | --- |
|  |  | **0h** | **+4h & MSC** | **p-value** | **0h** | **+4h & MSC** | **p-value** |
| 44 | CD7+CD11b+CD127+Tigit+ Treg | 1724  (134-16310) | 810  (93-22208) | ns | 1369  (0-3822) | 832  (0-4345) | ns |
| 45 | Treg | 1607  (416-9603) | 1204  (464-9680) | ns | 1436  (497-5591) | 1324  (208-6920) | ns |
| 46 | CD7+Treg | 2761  (485-23855) | 1517  (557-31318) | ns | 3578  (402-8998) | 2838  (830-13840) | ns |
| 47 | Tigit+ Treg | 4746  (1039-25988) | 2227  (1584-10924) | ns | 3162  (1470-24521) | 2358  (1014-6876) | 0.008 |
| 48 | CD161+PD-1+Tigit+ Treg | 2548  (375-7012) | 1612  (202-5979) | 0.012 | 1627  (435-7426) | 1270  (609-8748) | ns |

**Table S8. Treg**

| Subset | Cell type | Cells/µl  median (range)  week 25 | |  | Cells/µl  median (range)  week 26 | |  |
| --- | --- | --- | --- | --- | --- | --- | --- |
|  |  | **0h** | **+4h & MSC** | **p-value** | **0h** | **+4h & MSC** | **p-value** |
| 1 | NK cell | 16748  (3885-138479) | 5986  (928-88546) | 0.008 | 9289  (2446-35934) | 5996  (1958-22303) | 0.004 |
| 2 | Tigit+ NK cell | 2044  (369-32391) | 1062  (176-23631) | 0.016 | 1487  (218-12446) | 1234  (0-3311) | 0.039 |
| 3 | CD38+ NK cell | 9113  (4019-63284) | 5158  (1485-37410) | 0.023 | 6172  (1529-67499) | 3770  (1697-41092) | ns |
| 4 | CD38+CD39+ NK cell | 937  (157-19959) | 212  (0-12271) | 0.039 | 387  (0-28872) | 498  (0-14898) | ns |
| 5 | CD38+CD39+CD161+ NK cell | 1231  (187-24634) | 303  (0-6908) | 0.016 | 271  (0-25657) | 257  (0-9558) | ns |
| 6 | CD11c+ NK cell | 31913  (1607-64141) | 8482  (93-52790) | ns | 29004  (1366-70185) | 11178  (700-50958) | 0.004 |
| 7 | CD11c+CD38+NK cell | 10898  (624-23828) | 1151  (186-16990) | 0.016 | 6522  (1615-19156) | 1750  (0-13056) | 0.004 |
| 8 | CD11c+CD38+CD39+ NK cell | 10098  (670-64139) | 2641  (186-46389) | 0.008 | 9688  (621-81605) | 2534  (0-34380) | 0.004 |
| 9 | CD11c+CD161+ NK cell | 7008  (520-8960) | 1215  (0-10619) | ns | 4018  (1005-9140) | 1205  (305-6465) | 0.027 |
| 10 | CD57+ NK cell | 919  (134-3277) | 1646  (93-13097) | 0.016 | 335  (0-3528) | 992  (131-3120) | ns |
| 11 | Proliferating CD11b+CD11c+CD38+CD39+CD57+ NK cell | 3353  (0-12249) | 9118  (557-22446) | 0.008 | 2306  (373-7848) | 3367  (280-13214) | ns |
| 12 | Proliferating CD11b+CD11c+CD38+CD39+ NK cell | 11845  (6832-30368) | 20243  (4270-48035) | 0.016 | 8364  (2228-18457) | 14616  (4047-31924) | 0.008 |
| 13 | Proliferating CD11c+CD38+CD39+ NK cell | 1247  (268-7277) | 854  (0-16461) | ns | 1472  (98-17402) | 1098  (0-16699) | 0.039 |
| 14 | Proliferating CD11c+CD38+ NK cell | 2667  (0-4572) | 1053  (93-5764) | ns | 3384  (497-8451) | 1434  (140-4310) | 0.004 |

**Table S9. NK cells**
