# Supplementary figures and images for: Allogenic MSC infusion in kidney transplantation recipients promotes within 4 hours distinct B cell and T cell phenotypes
Source: Front Immunol. 2024 Oct 9;15:1455300. doi: 10.3389/fimmu.2024.1455300 (PMC11500071; doi:10.3389/fimmu.2024.1455300)

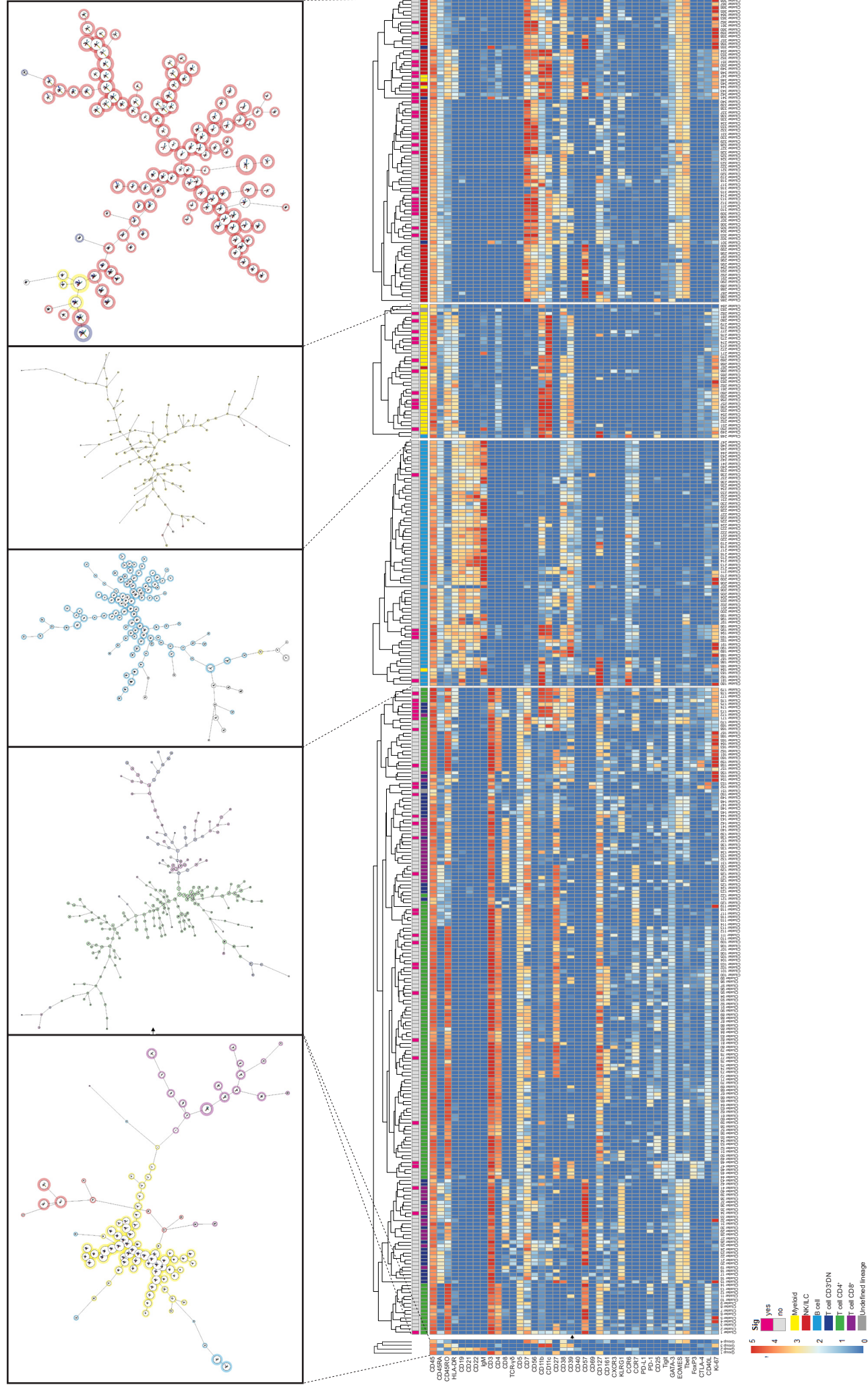

Figure S1. Heatmap. FlowSOM clusters and corresponding expression heatmap.

Supplement: Supplementary file 1 [file DataSheet1.zip › Figure S1.PDF]
