# Supplementary material for: Allogenic MSC infusion in kidney transplantation recipients promotes within 4 hours distinct B cell and T cell phenotypes
Source: Front Immunol. 2024 Oct 9;15:1455300. doi: 10.3389/fimmu.2024.1455300 (PMC11500071; doi:10.3389/fimmu.2024.1455300)
Supplement: Supplementary file 1 [file Figure S2.PDF › Figure S2.PDF]

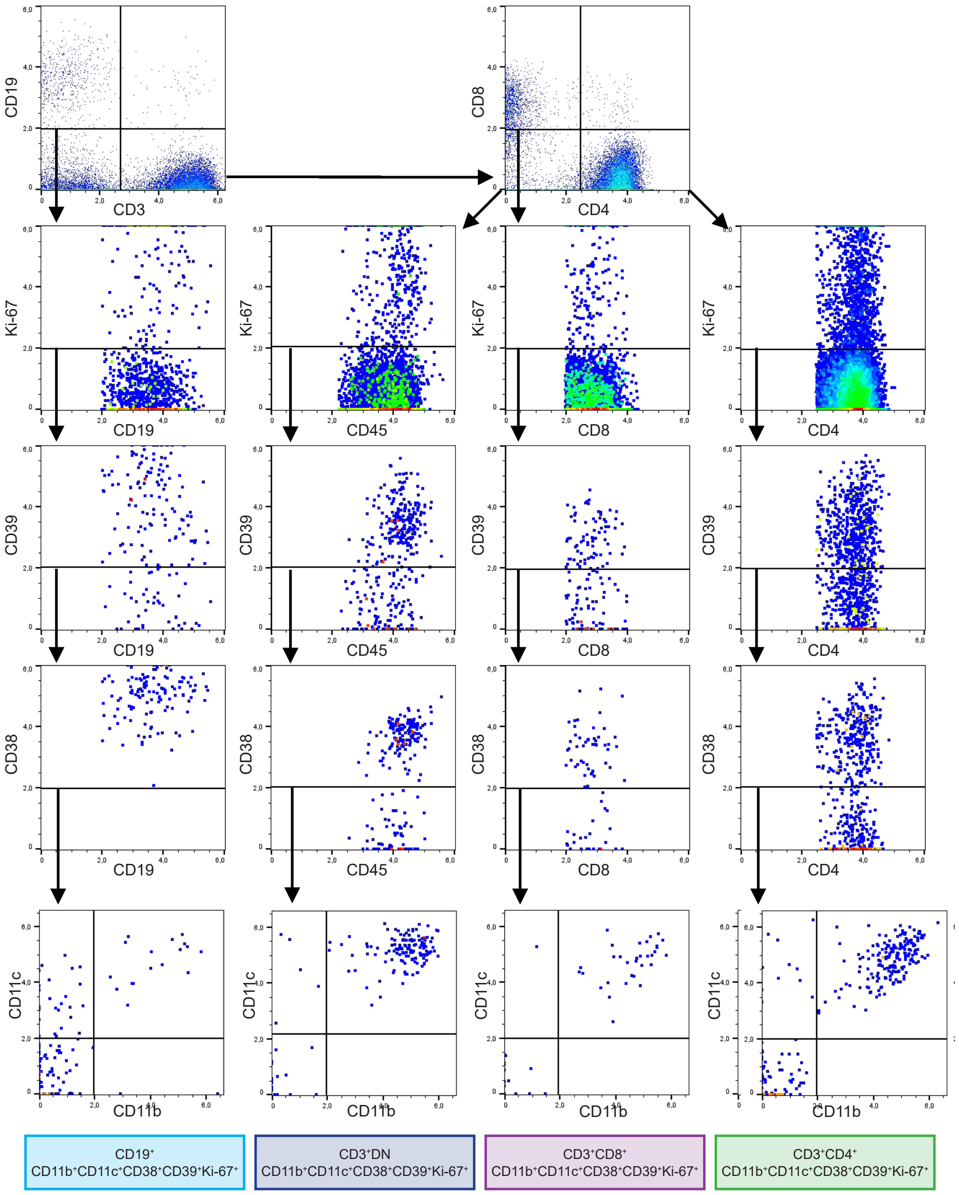

Figure S2. **Manual gating of CD11b^{+}CD11c^{+}CD38^{+}CD39^{+}Ki-67^{+} subsets.**
